# Robust Multi-Agent Path Finding Method for Obstacles and Environmental Changes in Factory Environments

**DOI:** 10.3390/s26134139

**Published:** 2026-07-01

**Authors:** Seihoon Park, Jinwon Lee, Geonhyeok Park, Ikhyeon Cho, Seongjoon Moon, Woojin Chung

**Affiliations:** Department of Mechanical Engineering, Korea University, Seoul 02841, Republic of Korea; yuiyt@korea.ac.kr (S.P.); jwlee0623@korea.ac.kr (J.L.); hyeok0913@korea.ac.kr (G.P.); tre0430@korea.ac.kr (I.C.); janismoon@korea.ac.kr (S.M.)

**Keywords:** multi-robot, multi-agent path finding, obstacle, robust planning, dynamic environment, delay, uncertainty

## Abstract

Multi-Agent Path Finding (MAPF) is a core technology for logistics automation in factories and warehouses. Guidance-based approaches that reflect the structural properties of factory environments have been widely adopted for computational efficiency and execution feasibility. However, these approaches generally assume static environments in which predefined guidance policies remain valid. Therefore, unexpected obstacles can cause inter-robot collisions or deadlocks. To make nominal MAPF plans robust against execution uncertainty, prior studies have incorporated bounded execution delays into MAPF. A representative method is *k* Robust Multi-Agent Path Finding (*k*R-MAPF), which models allowable execution delay using a global robustness parameter *k*. However, when large obstacle-induced delays are represented by a single global robustness parameter, *k*R-MAPF imposes unnecessary conservatism and increases the search space. This increase in search space raises planning runtime and reduces path efficiency in large-scale robot fleet operation. This paper proposes a multi-robot path planning framework that updates guidance policies for each segment based on real-time obstacle information. The proposed framework identifies robots affected by obstacles and selectively replans their paths, thereby reducing unnecessary computation while maintaining path planning success and path efficiency. Simulation results in a 100m×100m factory environment with up to 100 robots demonstrate that the proposed framework maintains a 100% success rate under all tested conditions. Compared with *k*R-MAPF with different values of *k*, the proposed framework reduces planning runtime by approximately 35–79% and flowtime by approximately 7–24%. These results demonstrate that obstacle-aware selective replanning can improve both real-time performance and path efficiency in dynamic factory environments. The proposed framework provides a technical basis for stable large-scale multi-robot operation in structured industrial environments.

## 1. Introduction

Multi-Agent Path Finding (MAPF) computes collision-free paths to agents on a graph. MAPF is a fundamental problem that underpins modern multi-robot coordination [1]. In smart factories and automated warehouses, path planning must coordinate hundreds of autonomous mobile robots in real time. These factory and warehouse environments require efficient transport operations while preventing collisions between robots [2,3].

The design of the MAPF algorithm generally requires a trade-off between the quality of the solution and the computation time. Finding an optimal solution is known to be NP-hard [4]. Consequently, previous studies have investigated various approaches beyond the optimal path search in large-scale environments. Such approaches include fast search methods, continuous-time models, and methods that account for robot dynamics to improve the execution feasibility. These studies have established important foundations for improving the quality of the solution [5,6], computational efficiency [7,8] and applicability in the real world [9,10] in MAPF.

However, collision-free discrete path planning alone is insufficient for practical multi-robot operation in factory environments [11]. Guidance-based approaches have been studied to incorporate robot navigation behavior into the path-planning stage [12,13,14]. These guidance-based approaches support the stable navigation of multiple robots in real-world environments. Factory layouts typically consist of structured workspaces organized around corridors and junctions. Therefore, path planning must account for segment-level navigation behavior. To address this requirement, a previous study abstracted the factory environment as a graph composed of corridors and junctions. Motion guidance tailored to the robot specifications was generated in advance for each segment and then executable paths were planned based on the generated motion guidance [14]. Reflecting the structural properties of factory environments in the path planning, the approach effectively organizes the search space. The approach also improves real-time multi-robot operation in large-scale environments. Therefore, guidance-based planning can be regarded as a representative environment representation framework for industrial path planning.

Guidance-based approaches can achieve high efficiency in static environments where predefined guidance policies remain valid. However, dynamic environments with frequent unexpected external obstacles make it difficult to maintain collision-free and deadlock-free operation. Consequently, robust MAPF methods have been studied to proactively incorporate execution uncertainty into the planning stage [15,16,17,18,19]. Among these methods, *k*R-MAPF is often used as a representative approach [15]. *k*R-MAPF addresses agent-level execution uncertainty, including localization errors, communication delays, and navigation delays. This method assigns a uniform temporal margin across the planned paths in the planning stage. The assigned margin prevents inter-robot collisions even under delayed execution. In obstacle-rich environments, temporary stops or delays caused by local obstacle avoidance can also be interpreted as execution delays. Under this interpretation, *k*R-MAPF can maintain stable execution against a bounded range of unexpected delays without requiring replanning.

The assignment of a uniform temporal margin globally to all agents and path segments is effective in ensuring system-wide safety. However, in factory environments where obstacles occur locally, the uniform temporal margin of *k*R-MAPF can produce conservative paths. Such conservatism imposes unnecessary waiting or detours even in path segments unaffected by obstacles, thereby reducing the throughput of the overall system. Moreover, overly conservative constraints generate excessive conflict tree branching during path search. The enlarged search space consequently degrades real-time performance in large-scale environments and can cause planning failures.

The present study proposes a multi-robot path planning method that prevents inter-robot collisions and deadlocks when obstacles appear or environmental changes occur. The proposed method maintains path efficiency while achieving low runtime. Unlike conventional conservative planning methods that uniformly impose delays across all robots and path segments, the proposed framework selectively incorporates obstacle information observed during robot navigation into global path planning. For this purpose, the proposed framework continuously maintains segment-level obstacle information detected by robots during execution. Based on the representation of the existing corridor and junction-based environment, the framework dynamically updates the guidance policies according to the type of obstacle in each segment. The framework also performs selective replanning only for robots affected by obstacles or environmental changes. Selective replanning reduces unnecessary computation while supporting an effective response to dynamic changes in factory environments.

Simulation experiments were conducted to validate the proposed method in a 100 m × 100 m factory environment, where 10 to 100 robots operate simultaneously. The proposed method maintained a 100% success rate in all experimental conditions. Compared to *k*R-MAPF, which considers different delay levels, the proposed method reduces planning runtime by approximately 35–79%. The proposed method also reduced overall flowtime by approximately 7–24%. These experimental results demonstrate that the proposed method improves computational performance while maintaining path efficiency.

The main contributions of this paper are summarized as follows.

1. We augment the guidance-based environment representation of [14] with a segment-level obstacle map maintained from runtime observations.
2. We formulate obstacle-aware guidance policies per segment, comprising motion-guidance updates and Safe Intervals for static obstacles, and a variable Traffic Regulation Margin for dynamic obstacles.
3. We integrate these policies into a CBS-based planner and replan only the robots expected to interact with affected segments, reducing unnecessary constraint generation.
4. We validate the framework in a 100 m × 100 m factory environment with up to 100 robots, against CBS and kR-MAPF baselines.

## 2. Related Works

### 2.1. Guidance-Based Multi-Agent Path Finding

Multi-Agent Path Finding is defined as the problem of planning collision-free paths for all agents, given the start and goal locations of all agents. In general, the path of each agent is represented as a sequence of move or wait actions over discrete time steps in a discrete workspace. The discrete representation enables clear collision definitions and efficient path search. However, discrete models do not sufficiently capture the continuous space and time in which real robots operate [4]. As a result, planned paths may be collision-free under discrete collision definitions, while still differing from actual robot navigation behaviors and interactions. Therefore, discrete path planning cannot fully describe realistic multi-robot operation.

To address the limitations of discrete modeling, approaches that consider continuous space and time have also been proposed [9]. However, the search space expands rapidly as the size of the environment and the degrees of freedom increase. The expanded search space increases planning runtime and makes optimal path search more difficult. The computational burden becomes more severe in environments where multiple robots operate simultaneously. Therefore, conventional discrete-space and continuous-space approaches involve a trade-off between execution feasibility and computational efficiency. A new approach is required to jointly consider both aspects for practical multi-robot operation in factory environments.

**Figure 1:**
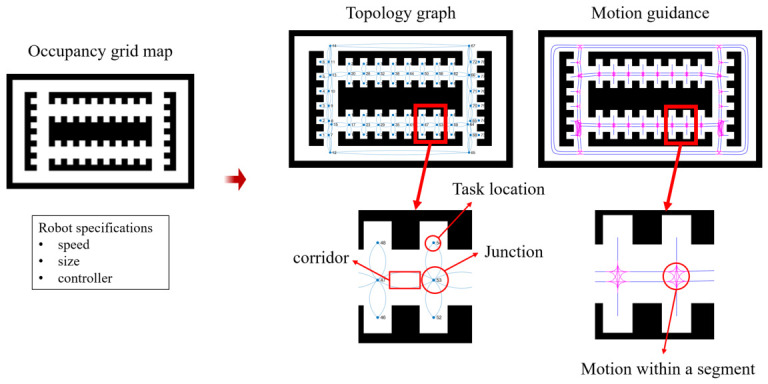
Guidance-based representation method for factory environments. The occupancy grid map is converted into a topology graph consisting of task locations, corridors, and junctions, where numbers denote graph nodes and lines represent their connections. Robot movements along each connection are managed using the corresponding motion guidance.

To address these limitations, a guidance-based path planning method was proposed, as shown in Figure 1. The guidance-based method reflects the structural properties of factory environments and robot specifications [14]. The method models a factory environment as a graph structure composed of junctions and corridors. In this representation, predefined motion guidance describes the navigation rules and behaviors that robots should follow in each segment. Consequently, path planning can consider both collision avoidance and segment-level navigation behavior.

Guidance-based formulation can improve computational efficiency by constraining the search space based on structural properties of factory environments. The formulation also supports executable path planning by incorporating robot specifications into the planning process. In addition, explicit movement rules for each segment facilitate stable management of inter-robot interactions. Therefore, guidance-based planning has been used as an effective and practical path planning method for multi-robot operation in factory environments.

However, a guidance-based approach is effective only when predefined guidance policies remain valid. When environmental changes or unexpected obstacles appear, predefined guidance policies may no longer be directly applicable.

### 2.2. Multi-Robot Operation Framework

**Figure 2:**
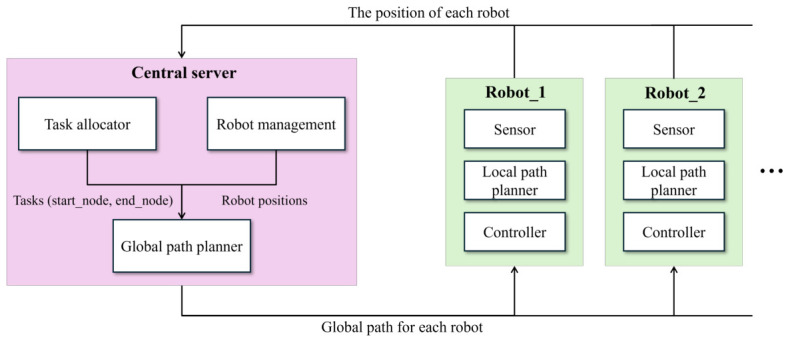
Multi-robot operation framework consisting of a central server and robots. The ellipsis indicates additional robots that can be included in the framework, while only two robots are illustrated for simplicity.

Multi-robot systems are generally operated through a hierarchical structure composed of a central server and multiple robots, as shown in Figure 2 [20,21,22,23]. The central server plans the overall paths based on the start and goal locations of the robot. The central server is also responsible for global path planning to prevent inter-robot collisions. In contrast, individual robots perform motion control during actual navigation. Individual robots also handle real-time responses, including local obstacle avoidance, based on sensor-based perception of the environment.

Within the hierarchical structure, robots perceive their surroundings during navigation and perform local obstacle avoidance in response to unexpected obstacles. However, local obstacle avoidance can cause discrepancies between the actual robot motion and the plans previously generated by the global path planner on the central server [21,22].

To address delays caused by local obstacle avoidance, the global path planner can incorporate delays into path planning to prevent inter-robot collisions and deadlocks [20]. In addition, when environmental changes or sensor-based local obstacle avoidance alter the planned paths, online replanning based on the current state is required. Therefore, a path planning method that maintains stable operation under plan changes caused by obstacle avoidance or environmental changes is necessary.

### 2.3. Multi-Agent Path Finding with Execution Uncertainty

In multi-robot systems, precomputed paths may no longer remain valid as originally planned because of execution delays or unexpected environmental changes during actual navigation. These execution uncertainties affect inter-robot interactions and cause discrepancies between global path plans and actual robot navigation. To address such uncertainty, previous studies have proposed approaches that plan paths that are robust to bounded execution delays [15,16,17,18,19].

Among these approaches, *k*R-MAPF [15] is a representative method that plans paths to ensure collision avoidance even when each robot is delayed by up to *k* time steps. The parameter *k* denotes the allowable range of execution delays for each robot, and larger values of *k* lead to paths that account for the higher level of delay. In this formulation, a path is considered k-robust if no collision occurs under any combination of robot delays within the k-step bound. Therefore, kR-MAPF expands the collision-checking condition from a single planned time step to a temporal interval that covers possible delayed arrivals. This allows the planner to proactively account for bounded execution uncertainty before the paths are executed.

**Figure 3:**
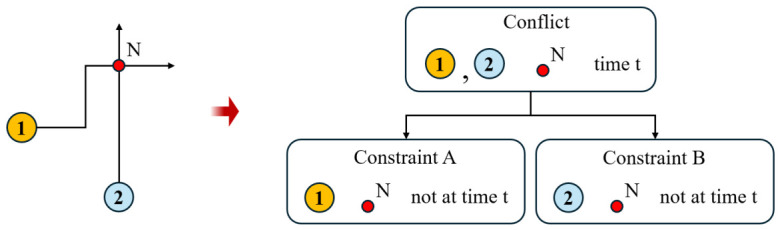
Conflict tree of CBS. When robots 1 and 2 collide at node *N* at time *t*, CBS branches the conflict tree into two child nodes by adding constraints 〈1,N,t〉 and 〈2,N,t〉, each of which prevents the corresponding robot from occupying node *N* at time *t*.

*k*R-MAPF is generally implemented within the Conflict-Based Search (CBS) framework using a conflict tree, as shown in Figure 3 [4]. At the high level of CBS, each node represents a specific set of constraints. Conventional CBS identifies conflict based on simultaneous node occupancy or simultaneous edge traversal. When a conflict occurs, the Conflict Tree is expanded by adding constraints that resolve the conflict. In contrast, *k*R-MAPF assumes that the movement of each robot can be delayed by up to *k* time steps. Based on this assumption, *k*R-MAPF extends the conflict definition to include possible occupation within a certain time interval. As a result, a single conflict is evaluated over a wider temporal range. The Conflict Tree then generates more branches, which enlarges the search space.

In addition, *k*R-MAPF plans paths by applying the same delay bound to all robots and all path segments. Therefore, conservative constraints can be imposed even on segments that are not actually affected by delays. In other words, the same temporal margin is enforced even in obstacle-free segments. As a result, the path flexibility decreases and robots may experience unnecessary waiting or detours. These conservative constraints also increase Conflict Tree branching, thereby expanding the search space and increasing planning cost. In environments where many robots operate simultaneously, the increased computational burden can degrade computational efficiency.

## 3. Methodology

### 3.1. Overall Framework

The proposed framework is built on the guidance-based environment representation introduced in [13]. To ensure real-time responsiveness, the framework is implemented as an event-driven architecture where the replanning process is triggered only when a robot detects a new obstacle or a significant change in an existing obstacle within its sensing range (10 m). To mitigate individual sensor limitations, such as occlusions, the framework aggregates real-time observations from the entire robot fleet into a centralized obstacle map, ensuring overall operational robustness. This paper does not modify the underlying topological representation itself. Instead, we introduce an obstacle-aware planning layer that updates segment-level guidance policies and replans only the paths of robots affected by observed obstacles. The framework is implemented within a CBS-based hierarchical search structure to balance robustness against obstacle-induced execution uncertainty and computational efficiency.

**Figure 4:**
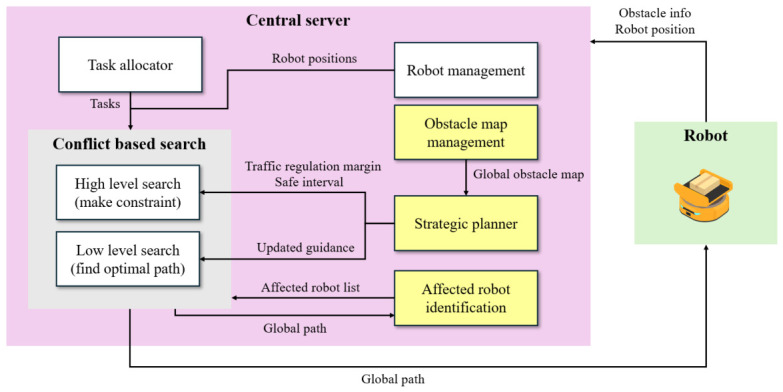
Overview of the proposed method.

As shown in Figure 4, the proposed framework introduces three core mechanisms. These mechanisms are implemented through the following module configuration.

The first module is Obstacle Map Management. This module collects real-time information observed by robots during navigation on the central server. Obstacle Map Management then updates and manages segment-level obstacle information through a global obstacle map M(t), where each segment is denoted by *s*.

The second module is the Strategic Planner. Based on the global obstacle map M(t), the Strategic Planner establishes obstacle-aware guidance policies for each segment. These policies include motion guidance updates, the Safe Interval SI(s), and the Traffic Regulation Margin Δ(s), which are determined according to the obstacle information in each segment. The resulting guidance and temporal regulation parameters are delivered to regulate inter-robot interactions during path planning.

The third module is Affected-Robot Identification. This module identifies affected robots $R_{affected}$, which are scheduled to pass through segments containing observed obstacles within a specified time range. The identified robot list is then sent to the global path planning module for replanning.

These three modules are integrated into the hierarchical structure of CBS. At the high level, extended conflict constraints are generated based on SI(s) and Δ(s) computed by the Strategic Planner. The high-level search then explores a valid combination of constraints. At the low level, the planner searches for a path for each robot by reflecting the modified guidance information. Through this process, the proposed framework plans a collision-free set of paths even in environments with dynamic obstacles.

This design selectively adjusts the degree of robustness according to the presence and location of obstacles. Consequently, the proposed framework responds to obstacles while maintaining path efficiency. The following sections describe the detailed mechanisms and design principles of the three core modules: Obstacle Map Management in Section 3.2, Strategic Planning in Section 3.3, and Affected-Robot Identification with selective replanning in Section 3.4.

### 3.2. Obstacle Representation and Management

**Figure 5:**
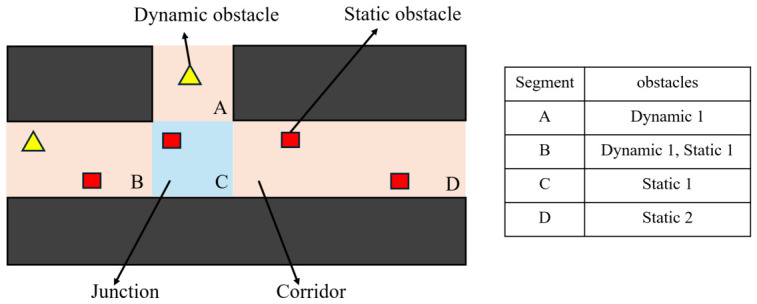
Segment-level obstacle management. The red square represents a static obstacle, and the yellow triangle represents a dynamic obstacle.

The proposed framework represents factory environments at the segment level based on the previously proposed guidance-based environment representation. As shown in Figure 5, the segment-level representation efficiently describes structured environments composed of corridors and junctions. The proposed framework manages obstacle information at the segment level and constructs a global obstacle map M(t).

Obstacles are classified into two types: static obstacles and dynamic obstacles. In particular, a dynamic obstacle that is temporarily stationary is handled in the same manner as other dynamic obstacles to ensure consistent planning.

Static obstacles refer to objects, such as boxes, that are not included in the prior map but appear at fixed locations. Static obstacles are represented using geometric information, including position and size. Geometric information is used to determine whether robots can traverse the corresponding segment under existing motion guidance. When a potential collision is expected, geometric information is also used to update the motion guidance.

In contrast, dynamic obstacles refer to moving objects, such as humans or forklifts. The proposed framework does not explicitly track the exact positions or sizes of dynamic obstacles. The absence of explicit trajectory tracking avoids reliance on uncertain long-term motion prediction and prevents unnecessary constraint generation. Instead, dynamic obstacles are modeled only on the basis of their presence in a specific segment at a given time. This presence-based representation reduces modeling complexity while preserving obstacle information required for path planning. Because one or more obstacles can exist in each segment simultaneously, the proposed framework defines and manages a set of obstacle segments O(s,t). The set of obstacles represents all obstacles determined to exist in segment *s* at time *t*. Based on this definition, the global obstacle map for the entire environment is constructed as $M\left(t\right)=\{O\left(s_{1},t\right),\ldots ,O\left(s_{N},t\right)\}$. The global obstacle map is centrally managed by the server.

The obstacle map is updated in real time based on the information observed by robots during navigation. Each robot detects obstacles within a certain sensing range and transmits the information to the server. The server updates the global obstacle map by integrating the received information. The aggregation of observations from individual robots enables the framework to reflect environmental changes in subsequent path planning.

This segment-based obstacle representation enables direct integration of obstacle information into guidance-based MAPF.

### 3.3. Strategic Planning for Guidance Policy Updates

In this study, guidance policies for each segment are defined based on the segment-based global obstacle map described in Section 3.2. Even when robots follow the same planned path, actual navigation behavior and inter-robot interactions can vary depending on the presence and type of obstacles in each segment. Therefore, appropriate guidance policies must be established for each segment according to the current obstacle condition. For this purpose, the proposed framework defines different guidance policies for static and dynamic obstacles.

**Figure 6:**
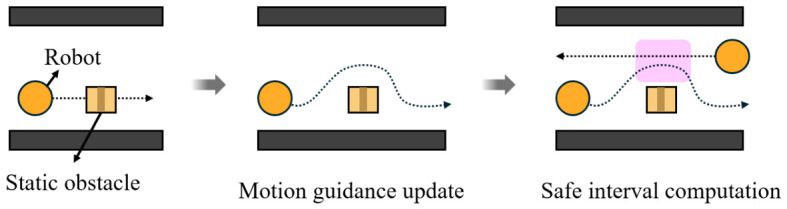
Guidance policies for handling static obstacles. The circle, arrow, and square represent the robot, its path, and a static obstacle, respectively. The pink shaded region indicates the conflict area.

Static obstacles are handled through two processes: motion guidance updates for obstacle avoidance and Safe Interval computation for inter-robot collision prevention, as shown in Figure 6. First, the motion guidance is updated for segments that contain static obstacles by reflecting the position and size of each obstacle. Based on the position of the static obstacle, a collision-avoidance trajectory is computed. The trajectory is then converted into a traversable motion form according to the robot specifications and the motion guidance is reconstructed accordingly. Using updated motion guidance, robots can traverse the corresponding segment while avoiding obstacles during path planning.

In addition to motion guidance updates for static obstacle avoidance, stable management of inter-robot interactions is required in narrow segments, such as corridors. Segments that allow bidirectional navigation require collision prevention between robots approaching from opposite directions. To address this requirement, the proposed framework applies the concept of Safe Intervals, which has been used in multi-robot path planning, to the guidance-based framework. In this study, the Safe Interval is defined as a set of available time intervals during which robots can enter the segment *s* without collisions. The Safe Interval is represented as the set of time intervals in which entry into a segment is allowed, as expressed in (1).

(1) $$SI\left(s\right)=\left\{\left[t_{i}^{start},t_{i}^{end}\right]|i=1,\ldots ,n\left(s\right)\right\}$$

To compute SI(s), the central server performs a temporal overlap analysis between bidirectional motion guidance policies predefined for each segment. First, the server simulates the expected occupancy time window for every potential entry time into segment *s* based on the robot’s kinematics. By comparing these occupancy windows for robots approaching from opposite directions, the system identifies specific entry time pairs that would lead to a physical conflict or a head-on deadlock within the narrow corridor. These conflicting periods are defined as ‘Unsafe Intervals.’ The algorithm then systematically subtracts these unsafe windows from the total planning horizon *T*, and the remaining non-conflicting time blocks are aggregated to form the set of SI(s). This refined process ensures navigation continuity by guaranteeing that any robot entering a segment during a Safe Interval will not encounter an opposing agent throughout its traversal. The computed SI(s) is used as a key constraint at the high level of CBS. During path planning, if the entry time $t_{enter}\left(r,s\right)$ of robot *r* into segment *s* is not included in SI(s), the case is regarded as an Interval Conflict. This conflict occurs when the entry time is in an unsafe interval between two consecutive safe intervals, such as $\left[t_{i}^{end},t_{i+1}^{start}\right]$.

When an Interval Conflict occurs, the high level of CBS generates a constraint to resolve the conflict. The constraint prohibits robot *r* from entering the segment *s* from the current entry time $t_{enter}$ to the start time of the next available interval $t_{i+1}^{start}$. Specifically, the constraint generated has the form $\left(r,s,\left[t_{enter},t_{i+1}^{start}\right]\right)$, which indicates that robot r cannot enter the segment before the next safe time. The low-level path search then satisfies the constraint by inserting a wait action into the previous segment and adjusting the entry time to after $t_{i+1}^{start}$. This process prevents deadlocks in corridors and supports navigation continuity.

**Figure 7:**
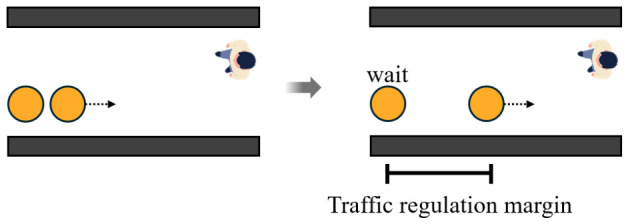
Guidance policies for handling dynamic obstacles. The circle and arrow represent the robot and its path, respectively.

For segments containing dynamic obstacles, a variable Traffic Regulation Margin Δ(s) is calculated according to the type and number of obstacles in each segment. Instead of assigning a fixed delay to all segments, the proposed framework establishes a temporal safety margin between robots when they enter obstacle-affected segments, as shown in Figure 7. The margin is determined on the basis of the obstacle condition in the corresponding segment.

In this study, a temporal margin between 0 and 5 s is calculated according to the type and number of obstacles, such as forklifts, hand carts, and workers. The Margin Conflict condition incorporating this margin is defined as shown in (2).

(2) $$\left|t_{enter}\left(r,s\right)-t_{enter}\left(r^{\prime },s\right)\right|<\Delta \left(s\right)$$

If the difference between the entry times of two robots is smaller than Δ(s), the high level of CBS determines the case as a potential Margin Conflict. The high-level search then generates additional constraints to enforce a sufficient temporal gap between robots.

This variable Traffic Regulation Margin reduces unnecessary conservatism caused by conventional *k*R-MAPF, which assumes a uniform delay k for all robots and path segments. Specifically, a large Δ(s) is assigned to segments with dense or high-risk obstacles to improve safety, while Δ(s) is minimized in segments with few obstacles. As a result, the proposed framework can improve both path efficiency, represented by flowtime, and the planning runtime of the overall system.

### 3.4. Affected Robot Identification and Selective Replanning

Dynamic obstacles move over time. Therefore, adjusting paths in advance based on future interactions with dynamic obstacles can cause discrepancies from actual situations. The Long-term prediction of dynamic obstacles involves uncertainty. Global replanning for all robots based on long-term obstacle prediction may therefore consider situations that do not actually occur. This process can generate unnecessary constraints and degrade planning efficiency and stability. Consequently, the proposed framework performs selective replanning only for robots that are likely to be affected by currently observed obstacles, instead of replanning paths for all robots.

To this end, the proposed framework determines the influence of obstacles based on the expected entry time of each robot into a specific segment along the planned path. When a new obstacle is observed, robots scheduled to pass through the obstacle-containing segment within a predefined time range of 20 s are defined as affected robots. This 20-s window is determined based on the average travel time required for a robot to traverse a single corridor segment (approximately 10 m at 0.5 m/s) in the tested factory layout. Specifically, for the expected entry time $t_{enter}\left(r,s\right)$ of robot *r* into segment *s*, robot *r* is classified as an affected robot if $t_{enter}\left(r,s\right)$ falls within 20 s of the current time. This criterion identifies only robots that are likely to interact with the observed obstacle.

For affected robots, paths are replanned by reflecting the guidance policies for each segment defined in Section 3.2. The replanning process jointly considers modified motion guidance and Safe Interval SI(s) for static obstacles. The process also considers constraints incorporating the Traffic Regulation Margin Δ(s) for dynamic obstacles. In contrast, robots unaffected by the observed obstacle maintain their existing paths, thus preventing unnecessary path changes. Selective replanning suppresses the expansion of the search space by limiting the generation of new constraints to the immediate vicinity of the obstacle. In the CBS framework, imposing constraints on all robots regardless of their proximity to the conflict increases the branching factor of the high-level Conflict Tree, leading to excessive search depth. By focusing only on robots within the 20-s window, we prevent the search tree from exploring unnecessary branches related to far-future interactions, thereby ensuring real-time responsiveness.

Consequently, the proposed selective replanning method adjusts paths only within the necessary range while considering uncertainty in dynamic environments. The proposed design reduces unnecessary constraint generation and enables appropriate responses to obstacles. As a result, the proposed framework improves both path planning efficiency and operational stability in multi-robot systems.

## 4. Experiments

### 4.1. Experimental Setup

**Figure 8:**
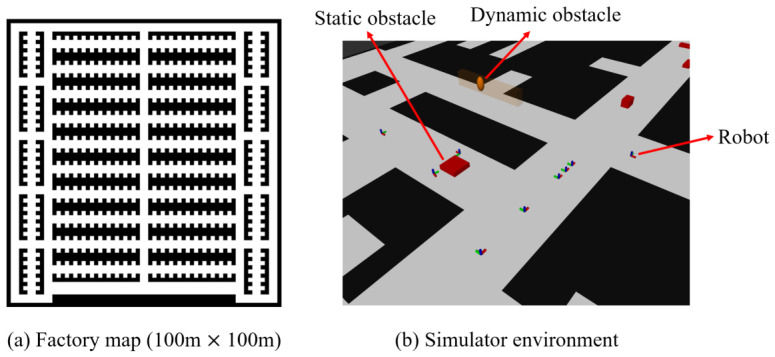
Simulation environment map: (**a**) warehouse environment of size 100m×100m; (**b**) visualization of robots and obstacles during the simulation process.

To evaluate the performance of the proposed method, simulations were conducted in a factory environment of 100m×100m, as shown in Figure 8. The experimental environment consists of corridors and junctions and is represented using a guidance-based environment representation. For each segment, motion guidance for robot navigation was generated in advance according to robot specifications. The proposed method incorporates graph-based obstacle information to update guidance policies and performs path planning and replanning accordingly.

The experiments were conducted by increasing the number of robots from 10 to 100. Each robot was modeled as a circular robot with a radius of 0.4 m, and its moving speed was set to 0.5 m/s. The initial tasks were generated by randomly assigning predefined task locations. When a robot completed its task, a new task was assigned online to move the robot to another task location. Obstacles were classified into static and dynamic obstacles. Static obstacles were placed to continuously occupy specific segments, whereas dynamic obstacles were represented on the basis of segment-level occupancy in corridors or junctions. When an obstacle existed in a specific segment, the corresponding segment was regarded as occupied. Dynamic obstacles were configured to move to adjacent segments over time. Each robot detected obstacles within a radius of 10 m during navigation. When a robot encountered an obstacle, a type-dependent delay of 0–5 s was applied during traversal of the corresponding segment. This specific range was determined to reflect typical transient delays caused by local obstacle avoidance, such as waiting for a passing forklift or a worker-commonly observed in actual industrial environments, allowing for flexible but robust fleet coordination.

All comparison methods were evaluated within the same guidance-based path planning framework. The environment representation, robot model, and task assignment method were kept identical. This experimental configuration allows performance differences among methods to be attributed to differences in path planning methods.

This study focuses on how the execution uncertainty caused by dynamic obstacles should be incorporated in the global path planning stage. Therefore, the performance of the proposed method was evaluated using two comparison methods.

First, CBS was used as a theoretical upper-bound baseline rather than a direct competitor. As an optimal solver, CBS provides a reference point to quantify the efficiency gap and computational overhead introduced by the obstacle-aware constraints in our proposed method.

In addition, *k*R-MAPF was selected as a representative baseline among prior approaches that considered execution uncertainty. *k*R-MAPF models execution delay using an allowable delay level k. The method prevents inter-robot collisions by incorporating the delay into constraints at the path planning stage. Because *k*R-MAPF directly corresponds to the delay caused by obstacle encounters addressed in this study, *k*R-MAPF provides an appropriate baseline for comparison with the proposed selective robustness assignment method. In this study, experiments were conducted by varying the allowed delay level *k* from 1 to 5 s.

Through this comparison setting, the proposed method was evaluated in terms of solution efficiency and computational efficiency in dynamic obstacle environments. The evaluation was conducted on the basis of the performance gap between ideal obstacle-free planning and conservative path planning methods that consider execution delays.

Performance evaluation was conducted to comprehensively assess planning stability, solution efficiency, and computational performance. The completeness of the planning was evaluated using the success rate within a 30 s time interval. The efficiency of the solution was measured using flowtime, defined as the sum of the execution times of the paths of all robots. In addition, the planning runtime and the proportion of cases within 1 s were measured to evaluate computational efficiency and real-time performance.

Each experiment was repeated 20 times, each 30-min trial involving multiple asynchronous replanning events triggered by dynamic obstacles.

### 4.2. Results

#### 4.2.1. Success Rate

**Figure 9:**
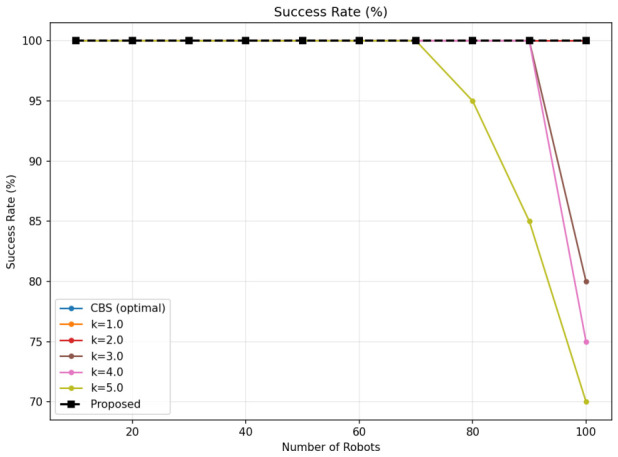
Success rate of path planning according to the number of robots. Overlapping curves indicate that the corresponding methods achieved the same success rate; some curves are hidden behind the proposed method curve when the success rate is 100%.

Figure 9 shows the path planning success rate according to the number of robots. The success rate was defined as the proportion of trials in which collision-free paths were planned for all robots within a 30-s timeout, which is a widely accepted standard in MAPF benchmarks for evaluating planning completeness within a reasonable real-time window.

The obstacle-free optimal path planning baseline maintained a 100% success rate under all experimental conditions. In contrast, *k*R-MAPF showed a decreasing success rate as the allowable delay level increased. The decrease became more pronounced as the number of robots increased. This result indicates that stronger delay-aware constraints make the search for valid solutions more difficult.

In contrast, the proposed method maintained a 100% success rate in all experiments, even with 100 robots. The 100% success rate demonstrates that the proposed method can stably plan paths even in environments with obstacles.

#### 4.2.2. Flowtime

**Figure 10:**
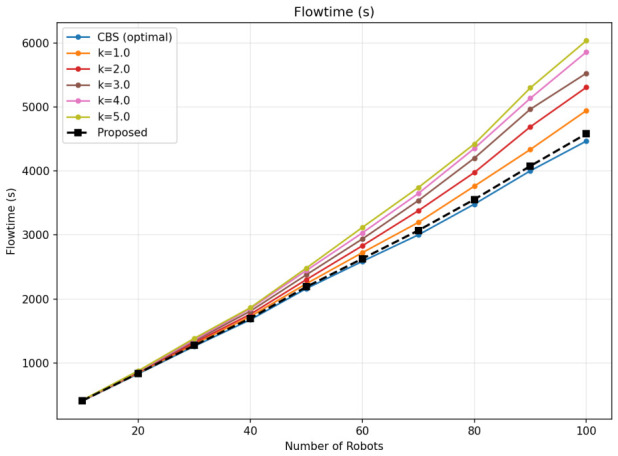
Flowtime according to the number of robots.

Figure 10 shows the flowtime, defined as the sum of the path execution times of all robots. Flowtime represents the execution time of the aggregate path required for the robots to reach their goal locations and is used as a metric to evaluate the throughput of the overall system.

The flowtime of the proposed method was measured as 4580.5 s, which corresponds to an approximately 24% reduction compared to *k*R-MAPF with k=5. In addition, the flowtime of the proposed method was only approximately 2.5% higher than the obstacle-free optimal path planning result of 4466.3 s. The flowtime results demonstrate that the proposed method maintains path efficiency while responding to obstacles.

#### 4.2.3. Path Planning Runtime

**Figure 11:**
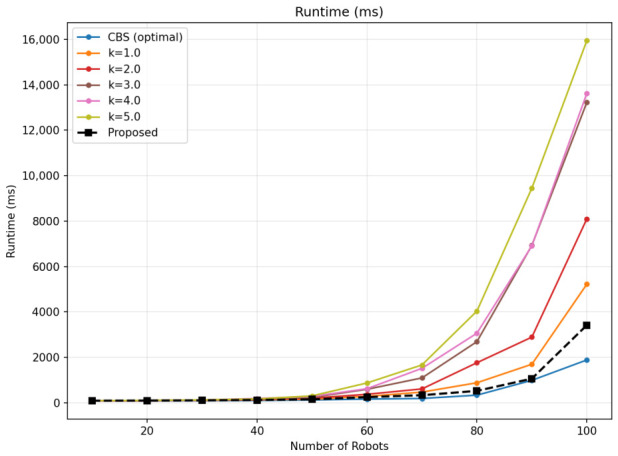
Path planning runtime according to the number of robots.

Figure 11 shows the path planning runtime according to the number of robots. In this evaluation, the runtime specifically represents the average computation time for the replanning stage triggered when obstacles are first detected within the environment.

For *k*R-MAPF, the runtime increased rapidly as the allowable delay level increased. In the 100-robot setting, *k*R-MAPF required approximately 5220.8 ms for k=1 and approximately 15,942.8 ms for k=5.

In contrast, the proposed method planned paths for all robots within an average runtime of approximately 3417.6 ms even in the 100-robot setting. The runtime corresponds to an approximately 79% reduction compared to *k*R-MAPF with k=5. The runtime results demonstrate that the proposed method reduces unnecessary search-space expansion by utilizing obstacle information.

#### 4.2.4. Real-Time Performance of Path Planning

**Figure 12:**
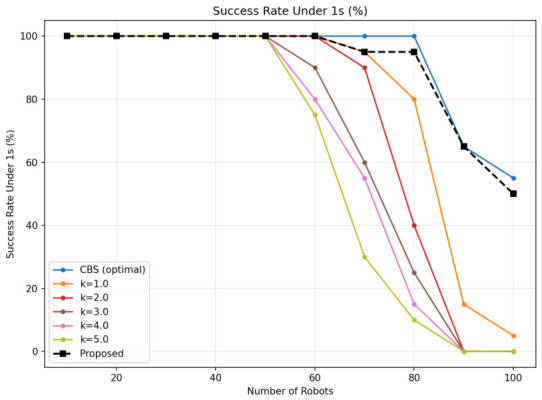
Success rate of path planning within 1 s according to the number of robots.

Figure 12 shows the ratio of trials in which path planning was completed within 1 s. This ratio was used to evaluate real-time performance.

For *k*R-MAPF, the trial solved within 1 s decreased rapidly as the allowable delay level increased. In the 100-robot setting, the ratio remained below 5% for all values of *k*.

In contrast, the proposed method completed the path planning in 1 s in 50% of the experiments under the same conditions. The real-time planning ratio of 50% is only 5 points lower than the ratio under obstacle-free conditions. The real-time performance results demonstrate that the proposed method limits the increase in computational complexity caused by obstacles and maintains real-time responsiveness close to that observed in obstacle-free environments.

### 4.3. Discussion

The performance improvement observed in this study comes from the structural difference in the generation and incorporation during path planning. The conventional *k*R-MAPF method assigns the same temporal margin to all robots and all path segments to handle possible execution delays. This uniform-margin approach is useful for maintaining stable collision avoidance. However, the broad generation of unnecessary constraints increases the branching of conflicts in CBS and substantially expands the search space. As the number of robots increases, the global constraints increase both the depth and width of the Conflict Tree, resulting in a rapid increase in runtime. This is because global constraints increase the branching factor of the CBS Conflict Tree by generating constraints even for robots and segments that are not directly affected by obstacles. In contrast, the proposed selective replanning limits constraint generation to affected robots and obstacle-related segments, thereby suppressing unnecessary branches and reducing the search space.

In contrast, the proposed method captures environmental changes through the Obstacle Map Management module and performs selective replanning only for affected robots and segments. By reducing unnecessary constraint generation, the proposed method limits the conflict candidates considered in CBS and reduces the search-space expansion. In other words, *k*R-MAPF incorporates potential delay situations in advance by applying a uniform temporal margin, whereas the proposed method assigns constraints only to segments affected by observed obstacles. Therefore, computation is performed only within the range required to resolve obstacle-induced conflicts. This constraint generation structure reduces computational complexity and leads to the runtime reduction observed in the experiments.

A similar mechanism also applies to path efficiency. The conventional *k*R-MAPF method plans conservative paths in all segments regardless of the presence of actual execution delays. Therefore, even in situations without actual delays, the planned paths tend to include unnecessary waiting or detours. Such conservative behavior increases the overall flowtime and reduces the throughput of the entire system in multi-robot environments. In contrast, the proposed method selectively adjusts the guidance policies for specific segments through the Strategic Planner. As a result, paths are modified only in segments where interactions with obstacles occur, whereas efficient existing paths are maintained in the remaining segments. This selective adjustment removes unnecessary conservatism while maintaining overall path efficiency. On the basis of this analysis, the proposed method has the following technical advantages over *k*R-MAPF.

First, the proposed method improves scalability. By applying selective constraints according to the type of obstacles present in each segment, rather than using uniform constraints, the proposed method reduces the number of branches in the high-level search. The reduction in branching mitigates the increase in computational cost, thereby supporting real-time performance even in large-scale multi-robot systems.

Second, the proposed method provides an advantage in terms of path efficiency. By responding locally only to segments where actual obstacles exist, the proposed method eliminates unnecessary conservatism in obstacle-free segments. This selective response prevents unnecessary waiting and detours by robots, maintains overall flowtime near the obstacle-free baseline, and can improve logistics performance in practical industrial applications.

Consequently, the integrated structure of Obstacle Map Management, Strategic Planning, and selective replanning mitigates the limitations of the conventional method, namely global conservatism and excessive search-space expansion. The proposed framework is significant because it provides an algorithmic framework that can improve both computational efficiency and path efficiency in multi-robot path planning under dynamic environments.

## 5. Conclusions

This study proposed a multi-robot path planning framework that utilizes obstacle information to improve robustness against execution uncertainty while maintaining real-time performance in factory environments with dynamic obstacles. The proposed method addresses the inefficiency of conventional robust planning by selectively managing segments containing obstacles based on obstacle information observed by robots during navigation.

The experimental results demonstrate that the proposed method maintained a 100% success rate in environments with up to 100 robots. Compared to conventional *k*R-MAPF, which considers execution delays, the proposed method reduced the planning runtime by up to approximately 79% and flowtime by up to approximately 24%. In addition, the proposed method completed the path planning within 1 s in 50% of the experiments. This real-time planning ratio is comparable to the 55% ratio achieved under obstacle-free conditions.

The experimental results demonstrate that the proposed method can respond to execution uncertainty while reducing unnecessary conservatism. This capability provides a technical advantage in balancing path efficiency, planning completeness, and real-time performance in multi-robot systems. Furthermore, the proposed approach is significant because it presents a framework for using obstacle information in the operation of multiple robots in structured workspaces, such as industrial environments.

Future work should extend the proposed framework by integrating prediction models that estimate changes in the obstacle state in advance, in addition to obstacle position and type. While this study validates the framework in a representative structured layout, applying the proposed method to a wider range of factory environments with diverse structural characteristics is necessary to further enhance its practical utility. Future research will focus on expanding these evaluations to ensure the framework’s robustness across various industrial settings.

## Data Availability

The original contributions presented in the study are included in the article, and further inquiries can be directed to the corresponding author.

## Abbreviations

The following abbreviations are used in this manuscript:

MAPF	Multi-Agent Path Finding
CBS	Conflict Based Search
*k*R-MAPF	*k* Robust Multi-Agent Path Finding
